# Case Report: Concurrent two adenomatoid tumors of liver

**DOI:** 10.3389/fonc.2025.1640018

**Published:** 2025-08-28

**Authors:** Lei Bi

**Affiliations:** ^1^ Department of Radiology, Shandong Provincial Hospital, Jinan, China; ^2^ Department of Radiology, Linyi People’s Hospital, Linyi, China

**Keywords:** adenomatoid tumor, liver, computed tomography, magnetic resonance imaging, case report

## Abstract

A 40-year-old man with two accidentally discovered subcapsular liver tumors was admitted to our hospital for further treatment. Computed tomography (CT) and magnetic resonance imaging (MRI) revealed two hypervascular lesions, which showed marked enhancement in arterial phase and prolonged enhancement in portal venous phase. The patient had not received any prior treatment, and the two lesions were pathologically confirmed after partial hepatectomy. Hematoxylin-eosin staining revealed a partially cystic, highly vascular, and well-encapsulated neoplasm. Immunohistochemistry findings demonstrated that the epithelioid tumor cells were positive for vimentin, calretinin, WT-1, cytokeratin, CD 31, CD 34, and D2-40, which supported their mesothelial origin. Immunohistochemistry for a mesothelial marker should be performed for determining the presence of an adenomatoid tumor when benign epithelioid cells are seen.

## Introduction

Adenomatoid tumors (AT) are benign and typically well-circumscribed neoplasms of mesothelial origin (1), which were first described by Golden and Ash (2). They mostly occur in the male and female genital tracts during a patient’s reproductive age. Extragenital adenomatoid tumors in adrenal gland (3), heart (4), mediastinum (5), liver (6–10), pancreas (11), peritoneum (12, 13), and pleura (14) have also been rarely reported. The tumors are usually discovered accidentally and are easy to be misdiagnosed by clinical and imaging examination.

We report a case of surgically confirmed liver adenomatoid tumors. To our knowledge, this is the first report of a case with two concurrent adenomatoid tumors in the liver.

## Case description

A 40-year-old man was admitted to the hospital for the accidentally discovered tumors of the liver. No obvious discomfort and remarkable previous medical history were reported. Physical examination was normal. Tumor marker levels, including carcinoembryonic antigen, carbohydrate antigen 19-9, carbohydrate antigen 125 and α-fetoprotein levels were within the normal range. The patient was negative for both the hepatitis B virus surface antigen and the hepatitis C virus antibody. No previous treatment was received. The patient denied any genetic basis, relevant family history or relationship to any other disease or syndrome. Contrast material-enhanced abdominal computed tomography (CT) revealed two relatively well-circumscribed oval lesions, both of which were on the segment VI. Magnetic resonance imaging (MRI) was then performed for further evaluation.

**Figure 1:**
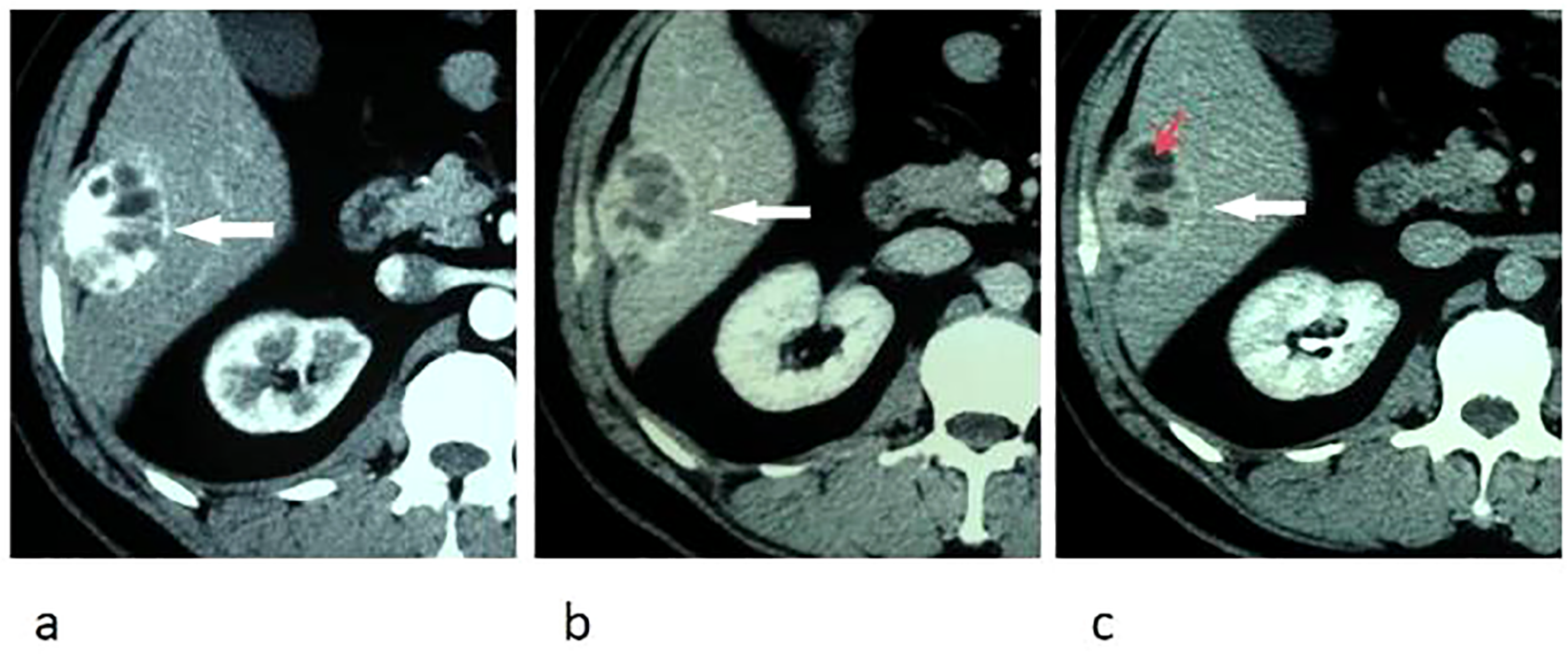
Transverse CT images of arterial phase **(a)**, portal venous phase **(b)**, and delay phase **(c)** show marked and prolonged enhancement of an oval liver mass, with unenhanced intratumoral cystic areas (red arrow) and enhanced capsule (white arrow).

On abdominal CT images, the larger mass was heterogeneous with some intratumoral multilobulated cysts, and the smaller nodule was homogeneous, measuring 4.5 cm and 1.5 cm correspondingly, both of which were closely related to the peritoneum. The tumors were hypervascular, demonstrating marked enhancement in arterial phase and prolonged enhancement in portal venous and delayed phases after intravenous injection of contrast material (**Figure 1**).

**Figure 2:**
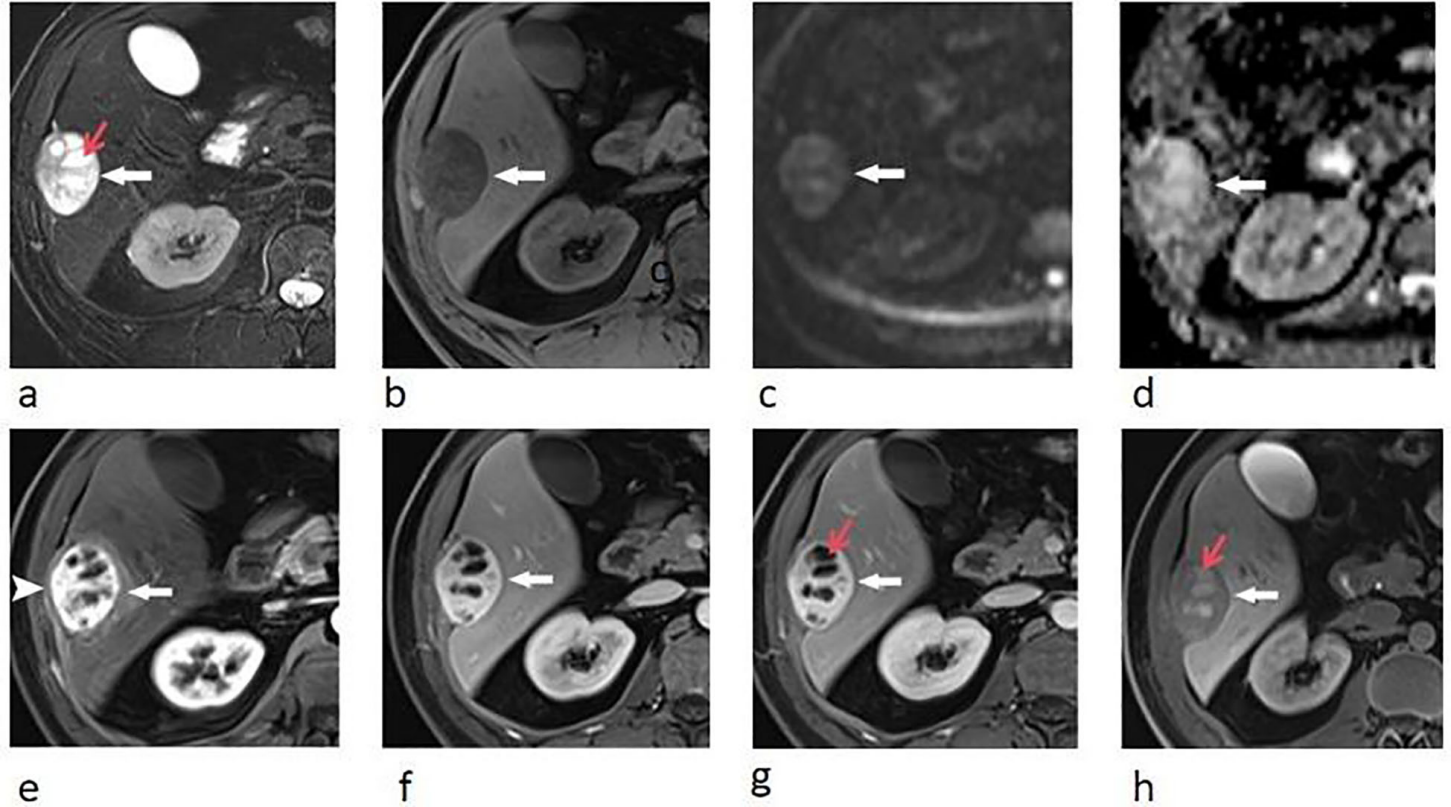
Transverse breath-hold turbo spin-echo T2-weighted image (repetition time msec/echo time msec, 3000/104) **(a)** and fat-suppressed T1-weighted volume interpolated body examination (VIBE) image (repetition time msec/echo time msec, 3.92/1.39) **(b)** shows a well-circumscribed liver mass with intratumoral cystic areas (red arrow). Diffusion-weighted image (repetition time msec/echo time msec, 4000/73, *b* = 800 s/mm^2^) **(c)** and apparent diffusion coefficient (ADC) **(d)** map show that the water mobility of the mass was slightly restricted. The water mobility of intratumoral cystic areas was not restricted. Transverse enhanced VIBE images of arterial phase **(e)**, portal venous phase **(f)**, and delay phase **(g)** show marked and prolonged enhancement of the liver mass (arrows), with unenhanced intratumoral cystic areas (red arrow). The liver capsule also showed enhancement (arrow head). In hepatobiliary phase **(h)**, the mass was hypointense compared with the liver parenchyma, with these cystic areas hyperintense (red arrow).

**Figure 3:**
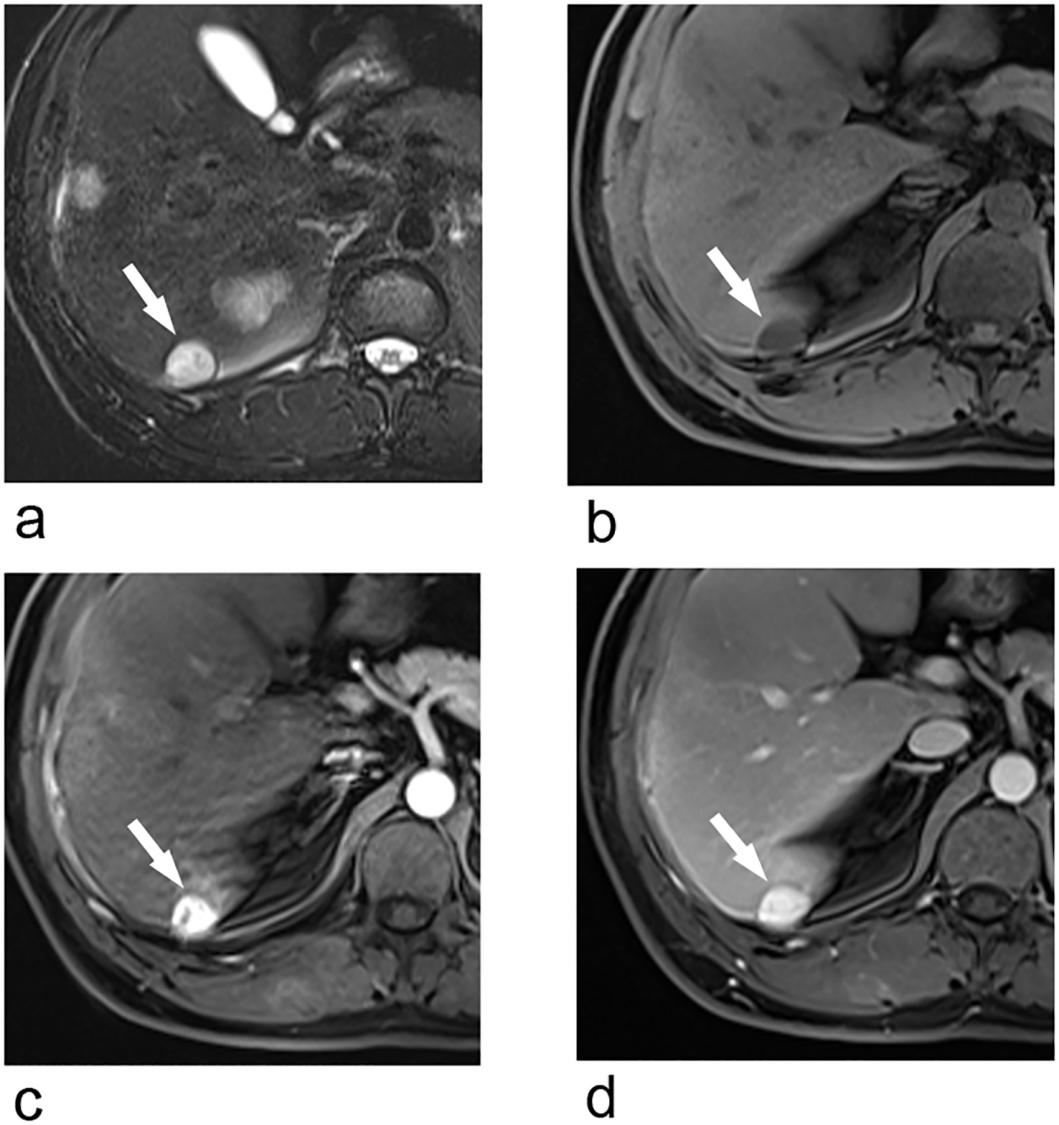
Transverse breath-hold turbo spin-echo T2-weighted image (repetition time msec/echo time msec, 3000/104) **(a)** and fat-suppressed T1-weighted volume interpolated body examination (VIBE) image (repetition time msec/echo time msec, 3.92/1.39) **(b)** shows a well-circumscribed liver nodule. Transverse arterial phase **(c)** and portal venous phase **(d)** show marked enhancement of the nodule.

Upper abdominal MRI (3.0T, Magnetom Verio, Siemens, Germany) was subsequently performed. The large liver mass was heterogeneous on MR images, with hypointense on T1-weighted images and hyperintense on T2-weighted images. There were multi-lobulated, cystic areas within the mass. After injection of gadoxetic acid (Gd-BOPTA, MultiHance, Shanghai Bracco Sine Pharmaceutical, China), the mass showed inhomogeneous hyperintense in arterial phase and revealed prolonged enhancement in the portal venous phase and delayed phase (3 min). In hepatobiliary phase, the mass was hypointense with the cystic areas hyperintense. In addition, the liver capsule and the adjacent peritoneum also showed obvious enhancement (**Figure 2**). The nodule was hypointense on T1-weighted imaging and hyperintense on T2-weighted imaging. It revealed marked enhancement in arterial phase, and showed prolonged enhancement in portal venous phase and delayed phase (**Figure 3**).

Given the uncertainty of the diagnosis and the patient’s anxiety about the tumors, the patient chose to undergo surgical treatment. The two liver lesions were resected in our hospital through partial hepatectomy of segment VI. After the surgery, the patient recovered well and expressed satisfaction about the treatment.

**Figure 4:**
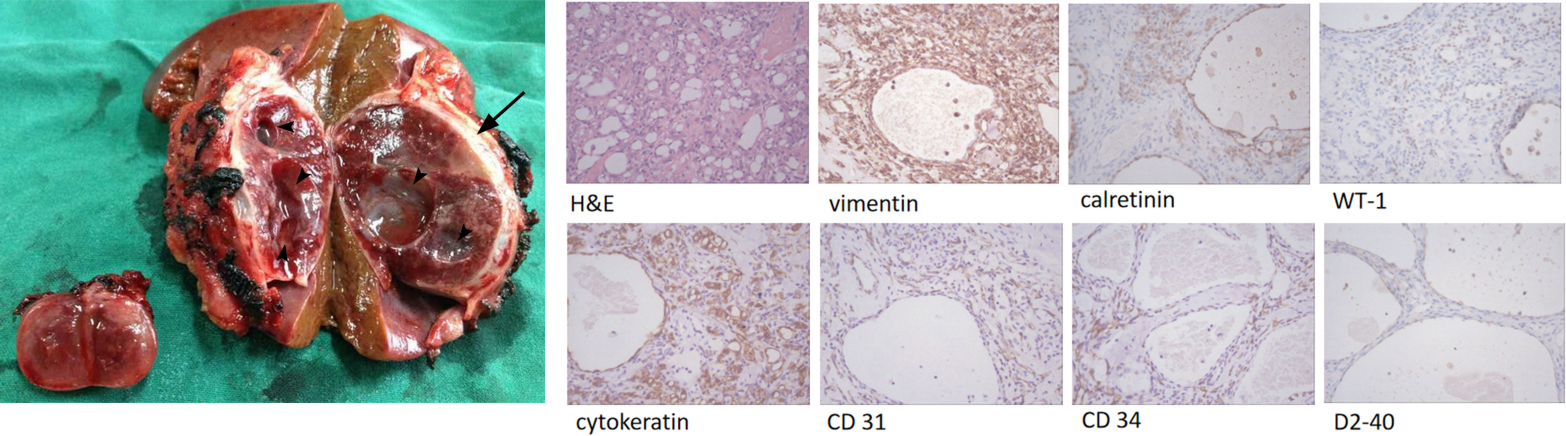
Gross pathologic specimen shows a heterogeneous well-circumscribed and encapsulated tumor (arrow) with multiple cystic areas (arrow heads), and a small well-circumscribed nodule. High-power-field view of the resected tumor specimen show a lesion with various-sized capillary anastomosis and fibrotic component (H&E). Immunohistochemistry shows positive for vimentin, calretinin, WT-1, cytokeratin, CD 31, CD 34, and D2-40.

Grossly, the tumors showed a hemorrhagic cut surface and was encapsulated with a complete capsule, the larger mass also showed obvious cystic structures (**Figure 4**). Hematoxylin-eosin staining revealed a partially cystic, highly vascular, and well-encapsulated neoplasm. Immunohistochemistry findings demonstrated that the epithelioid tumor cells were positive for vimentin, calretinin, WT-1, cytokeratin, CD 31, CD 34, and D2-40, which supported their mesothelial origin. The Ki-67 proliferation index was less than 5% (**Figure 4**).

**Figure 5:**
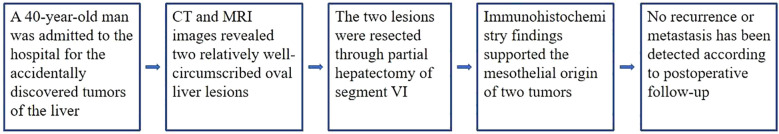
Clinical timeline of the patient.

Postoperative follow-up was conducted annually, and till the last follow-up, no recurrence or metastasis has been detected (**Figure 5**).

## Discussion

Adenomatoid tumors are benign uncommon neoplasms of mesothelial origin. Only five cases of hepatic adenomatoid tumor have been described in the medical literature (6–10). We report a case of two concurrent liver adenomatoid tumors, which has not been reported before.

Due to the different content of cystic spaces, smooth muscle, and fibrous tissue, adenomatoid tumors could show various image features, leading them difficult to be differentiated from other benign or malignant tumors on clinical CT or MRI examination (8). In this case, the preoperative imaging differential diagnosis of the liver mass included hepatocellular carcinoma (HCC), focal nodular hyperplasia (FNH), hepatic adenoma and hemangioma because of the hypervascular feature of the tumor in this patient (**Table 1**).

**Table 1:** Comparative summary of different liver tumors.

Tumor name	Tumor characteristics
Adenomatoid tumor	• relatively well-circumscribed • with intratumoral multilobulated cysts • hypervascular with marked enhancement in arterial phase and prolonged enhancement in portal venous and delayed phases • intratumoral multilobulated cysts are hyperintense in hepatobiliary phase
Hepatocellular carcinoma (HCC)	• α-fetoprotein level is higher than normal • most tumors show marked arterial enhancement and washout in portal venous phase • sclerosing hepatocellular carcinoma is extremely rare, frequently with extrahepatic metastasis • fibrolamellar hepatocellular carcinomas typically arise in non-cirrhotic livers in young adults, with a large central scar usually visible
Focal nodular hyperplasia (FNH)	• rarely encapsulated • strong enhancement in arterial phase with central non-enhancing scar • in hepatobiliary phase, FNH is isointense compared with the liver parenchyma with a hyperintense central star (typical)
Hepatic adenoma	• predominantly found in young females and associated with the use of contraceptives • internal necrosis or hemorrhage could be seen in large adenomas • hypervascular in arterial phase, and is generally isointense or hypointense in portal venous phase • hypointense in hepatobiliary phase
Hemangioma	• hypervascular enhancement and hypointense in hepatobiliary phase

HCC is the most common primary malignant tumor of the liver. It typically appears as a liver lesion with marked arterial enhancement and washout in portal venous phase. Although some variants of HCC may not show washout in portal venous phase, such as sclerosing and fibrolamellar hepatocellular carcinomas, which have abundant fibrous stroma and exhibit prolonged enhancement (15), they still should not taken into consideration because of the normal α-fetoprotein level. In addition, sclerosing hepatocellular carcinoma is extremely rare, frequently with extrahepatic metastasis in most cases. Fibrolamellar hepatocellular carcinomas typically arise in non-cirrhotic livers in young adults, with a large central scar usually visible (16). These findings were not present in this patient.

Focal nodular hyperplasia (FNH) is a proliferation of non-neoplastic hepatocytes that are abnormally arranged. It is rarely encapsulated. FNH often shows strong enhancement in arterial phase with central non-enhancing scar. In hepatobiliary phase, the tumor is isointense compared with the liver parenchyma with a hyperintense central star, which is typical on MRI. The central scar is a helpful distinguishing feature of FNH (although not specific) that can be seen in 78% of lesions (17, 18).

Hepatic adenoma is a rare monoclonal benign liver tumour, predominantly found in young females and associated with the use of contraceptives (19). It is formed by large plats or cord cells that are similar to hepatocytes (20). Internal necrosis or hemorrhage could be seen in large adenomas (17). Lesions often appear hypervascular in arterial phase, and are generally isointense or hypointense to the surrounding liver in portal venous phase. In hepatobiliary phase, it is hypointense because of the lack of normal hepatic cells which can uptake Gd-BOPTA. In addition, some hepatic adenomas containing fatty tissue will show signal attenuation on out-phase image, which was not seen in this case.

Atypical hemangiomas should carefully be differentiated. Liang C et al. has reported that some atypical hemangiomas could have cystic changes and even fluid-fluid level (21). Its hypervascular enhancement and hypointense in hepatobiliary phase could mimic the right diagnosis of this patient.

Hepatocyte specific contrast agents including gadoxetic acid (Gd-EOB-DTPA, Primovist, Bayer, Germany) and Gd-BOPTA were introduced in recent years. The hepatocytes with normal function which have the cloned organic anion transporting polypeptides (OATPs) specifically take up these agents, and excreted them by multidrug resistance-associated proteins (MRPs) to bile canaliculi (MRP2 = apical transporter) or sinusoidal space (MRP3, MRP4 = basolateral transporters) (22). Hepatobiliary phase (about 20 to 90 minutes later after intravenous administration of these agents) helps to differentiate malignant from benign lesions. In hepatobiliary phase, benign lesions with normally functioning hepatocytes will show iso- or hyperintense compared with liver parenchyma, and vice-versa.

Five cases of hepatic adenomatoid tumor have been described in the medical literature, and among them, only Kim JB et al. (8) mentioned the use of hepatocyte specific contrast agent. In his study, he used Gd-EOB-DTPA as the contrast agent. The tumor in their case had some similar multi-lobulated, cystic areas as seen in our case, but in our case, the cystic areas within the larger mass showed marked hyperintense in hepatobiliary phase, which was not reported in their study.

Gd-BOPTA has both the function of hepatocyte specific contrast agent and tissue interstitial contrast agent. Therefore, in tissues with abundant fibrous structures, Gd-BOPTA can remain in the tissue interstitial space for a longer period of time. It is a well-established phenomenon that the tumor with abundant fibrous stroma could reveal persistent enhancement up to 4h after administration of gadolinium chelates (23). Hematoxylin-eosin staining revealed that the lesion in our case has a large fibrotic component. So we hypothesized that the delayed enhancement of these cystic areas may be because that the interstitial space could contain contrast agent for a long time.

In summary, the diagnosis of liver adenomatoid tumor is challenging due to its rarity and the similar manifestation of other neoplasms. Immunohistochemistry analysis enabled us to identify tumor cells of mesothelial origin. Hypervascular and multi-lobulated, cystic areas are clues to the imaging diagnosis of them. In addition, in hepatobiliary phase, these cystic areas could show hyperintense when the lesion contains a large fibrotic component. Since this is the first case of two concurrent liver adenomatoid tumors, the etiology of dual tumor occurrence is still unclear. More researches are needed to explain this issue.

## Data Availability

The raw data supporting the conclusions of this article will be made available by the authors, without undue reservation.

## References

[1] Karpathiou G Hiroshima K Peoc’H M. Adenomatoid tumor: A review of pathology with focus on unusual presentations and sites, histogenesis, differential diagnosis, and molecular and clinical aspects with a historic overview of its description. Adv Anat Pathol. (2020) 27:394–407. doi: 10.1097/PAP.0000000000000278, PMID: 32769378

[2] Golden A Ash JE. Adenomatoid tumors of the genital tract. Am J Pathol. (1945) 21:63–79., PMID: 19970804 PMC1934086

[3] Guan J Zhao C Li H Zhang W Lin W Tang L. Adenomatoid tumor of the adrenal gland: report of two cases and review of the literature. Front Endocrinol (Lausanne). (2021) 12:692553. doi: 10.3389/fendo.2021.692553, PMID: 34248850 PMC8261242

[4] Natarajan S Luthringer DJ Fishbein MC. Adenomatoid tumor of the heart: report of a case. Am J Surg Pathol. (1997) 21:1378–80. doi: 10.1097/00000478-199711000-00014, PMID: 9351577

[5] Plaza JA Dominguez F Suster S. Cystic adenomatoid tumor of the mediastinum. Am J Surg Pathol. (2004) 28:132–8. doi: 10.1097/00000478-200401000-00016, PMID: 14707875

[6] Zhou R Wang HL. Adenomatoid tumor of the liver with prominent admixed cavernous hemangioma-like vessels: an exceptional occurrence. Int J Surg Pathol. (2025) 33:1505–12. doi: 10.1177/10668969251323930, PMID: 40080866 PMC12276407

[7] Adachi S Yanagawa T Furumoto A Fujino S Doi R Dono K. Adenomatoid tumor of the liver. Pathol Int. (2012) 62:153–4. doi: 10.1111/j.1440-1827.2011.02767.x, PMID: 22243787

[8] Kim JB Yu E Shim JH Song GW Kim GU Jin YJ. Concurrent hepatic adenomatoid tumor and hepatic hemangioma: a case report. Clin Mol Hepatol. (2012) 18:229–34. doi: 10.3350/cmh.2012.18.2.229, PMID: 22893875 PMC3415884

[9] Nagata S Aishima S Fukuzawa K Takagi H Yonemasu H Iwashita Y. Adenomatoid tumour of the liver. J Clin Pathol. (2008) 61:777–80. doi: 10.1136/jcp.2007.054684, PMID: 18505892 PMC2569191

[10] Hayes SJ Clark P Mathias R Formela L Vickers J Armstrong GR. Multiple adenomatoid tumours in the liver and peritoneum. J Clin Pathol. (2007) 60:722–4. doi: 10.1136/jcp.2005.035386, PMID: 17483249 PMC1955065

[11] Overstreet K Wixom C Shabaik A Bouvet M Herndier B. Adenomatoid tumor of the pancreas: a case report with comparison of histology and aspiration cytology. Mod Pathol. (2003) 16:613–7. doi: 10.1097/01.MP.0000072803.37527.C8, PMID: 12808068

[12] Wang LY Zhong YQ Li HG Zeng YJ Zhang SN Chen WX. Adenomatoid tumor of peritoneum–a case report. Zhonghua Yi Xue Za Zhi. (2005) 85:495–7., PMID: 15854561

[13] Hatano Y Hirose Y Matsunaga K Kito Y Yasuda I Moriwaki H. Combined adenomatoid tumor and well differentiated papillary mesothelioma of the omentum. Pathol Int. (2011) 61:681–5. doi: 10.1111/j.1440-1827.2011.02720.x, PMID: 22029681

[14] Minato H Nojima T Kurose N Kinoshita E. Adenomatoid tumor of the pleura. Pathol Int. (2009) 59:567–71. doi: 10.1111/j.1440-1827.2009.02407.x, PMID: 19627540

[15] Mittal PK Moreno CC Kalb B Mittal A Camacho JC Maddu K. Primary biliary tract Malignancies: MRI spectrum and mimics with histopathological correlation. Abdom Imaging. (2015) 40:1520–57. doi: 10.1007/s00261-014-0300-0, PMID: 25416002

[16] Soussan M Felden A Cyrta J Morere JF Douard R Wind P. Case 198: solitary fibrous tumor of the liver. Radiology. (2013) 269:304–8. doi: 10.1148/radiol.13121315, PMID: 24062563

[17] Jang H-J Yu H Kim TK. Imaging of focal liver lesions. Semin Roentgenology. (2009) 44:266–82. doi: 10.1053/j.ro.2009.05.008, PMID: 19715792

[18] Kamaya A Maturen KE Tye GA Liu YI Parti NN Desser TS. Hypervascular liver lesions. Semin Ultrasound CT MRI. (2009) 30:387–407. doi: 10.1053/j.sult.2009.06.001, PMID: 19842564

[19] Maillette de Buy Wenniger L Terpstra V Beuers U. Focal nodular hyperplasia and hepatic adenoma: epidemiology and pathology. Dig Surg. (2010) 27:24–31. doi: 10.1159/000268404, PMID: 20357448

[20] Palmucci S. Focal liver lesions detection and characterization: The advantages of gadoxetic acid-enhanced liver MRI. World J Hepatol. (2014) 6:477–85. doi: 10.4254/wjh.v6.i7.477, PMID: 25067999 PMC4110539

[21] Liang C Yu R Wang L Chen Y. Case series multilocular cystic hemangioma of the liver: Three cases and literature review. Med (Baltimore). (2024) 103:e39287. doi: 10.1097/MD.0000000000039287, PMID: 39151515 PMC11332750

[22] Jeong WK Kim YK Song KD Choi D Lim HK. The MR imaging diagnosis of liver diseases using gadoxetic acid: emphasis on hepatobiliary phase. Clin Mol Hepatol. (2013) 19:360–6. doi: 10.3350/cmh.2013.19.4.360, PMID: 24459639 PMC3894434

[23] Gabata T Matsui O Kadoya M Yoshikawa J Ueda K Kawamori Y. Delayed MR imaging of the liver: correlation of delayed enhancement of hepatic tumors and pathologic appearance. Abdominal Imaging. (1998) 23:309–13. doi: 10.1007/s002619900347, PMID: 9569304

